# Aphid endosymbiont facilitates virus transmission by modulating the volatile profile of host plants

**DOI:** 10.1186/s12870-021-02838-5

**Published:** 2021-01-29

**Authors:** Xiao-Bin Shi, Shuo Yan, Chi Zhang, Li-Min Zheng, Zhan-Hong Zhang, Shu-E Sun, Yang Gao, Xin-Qiu Tan, De-Yong Zhang, Xu-Guo Zhou

**Affiliations:** 1 Laboratory of Pest Management of Horticultural Crop of Hunan Province, Hunan Plant Protection Institute, Hunan Academy of Agricultural Sciences, Changsha, 410125 China; 2 Department of Entomology, University of Kentucky, Lexington, KY 40546 USA; 3 Institute of Vegetable, Hunan Academy of Agricultural Sciences, Changsha, 410125 China

**Keywords:** *Myzus persicae*, *Buchnera aphidicola*, Cucumber mosaic virus, Plant volatile, Multi-trophic interaction

## Abstract

**Background:**

Most plant viruses rely on vectors for their transmission and spread. One of the outstanding biological questions concerning the vector-pathogen-symbiont multi-trophic interactions is the potential involvement of vector symbionts in the virus transmission process. Here, we used a multi-factorial system containing a non-persistent plant virus, cucumber mosaic virus (CMV), its primary vector, green peach aphid, *Myzus persicae*, and the obligate endosymbiont, *Buchnera aphidicola* to explore this uncharted territory.

**Results:**

Based on our preliminary research, we hypothesized that aphid endosymbiont *B. aphidicola* can facilitate CMV transmission by modulating plant volatile profiles. Gene expression analyses demonstrated that CMV infection reduced *B. aphidicola* abundance in *M. persicae*, in which lower abundance of *B. aphidicola* was associated with a preference shift in aphids from infected to healthy plants. Volatile profile analyses confirmed that feeding by aphids with lower *B. aphidicola* titers reduced the production of attractants, while increased the emission of deterrents. As a result, *M. persicae* changed their feeding preference from infected to healthy plants.

**Conclusions:**

We conclude that CMV infection reduces the *B. aphidicola* abundance in *M. persicae*. When viruliferous aphids feed on host plants, dynamic changes in obligate symbionts lead to a shift in plant volatiles from attraction to avoidance, thereby switching insect vector’s feeding preference from infected to healthy plants.

## Background

Most of plant viruses are transmitted by arthropod vectors such as whiteflies and aphids. Plant viruses can directly or indirectly influence vector physiology and behavior to facilitate their transmission [1]. Viruses can also alter plant volatiles to recruit insect vectors for efficient transmission [2, 3]. To understand the mechanisms governing the virus transmission is important to reveal the plant-virus-vector co-evolution, and provide a basis for manipulating vectors to limit virus spread in plants [4].

Cucumber mosaic virus (CMV), genus Cucumovirus, family Bromoviridae, is one of the ten most devastating plant viruses [5, 6]. CMV can infect a broad range of hosts, including vegetables and ornamentals. CMV is vectored by over 80 aphid species in a non-persistent manner. Among those, *Myzus persicae* is the most extensively studied vector [7]. CMV-infected plants release a greater quantity of volatiles than healthy plants, and aphids were attracted to infected plants [8]. CMV infection can trigger antibiosis against aphids, leading to rapid aphid dispersal after virus acquisition [9].

*Buchnera aphidicola,* a primary symbiotic bacterium, provides essential amino acids for aphids. In turn, aphids offer *B. aphidicola* with nitrogenous substrates, including non-essential amino acids for the endosymbiont to produce essential amino acids [10]. It has been hypothesized that endosymbionts of insects contribute to the transmission of certain luteovirids and geminiviruses [11–13], although the evidences are debatable [14] . Nevertheless, the consensus is that additional research is needed to demonstrate the direct and/or indirect involvement/effect of endosymbionts in the transmission of plant virus in different vector-virus systems [12]. Up to now, the role of *B. aphidicola* in the transmission of non-persistent virus such as CMV is largely unclear.

During insect feeding, endosymbionts can alter the quantity and quality of plant volatiles to affect their insect hosts [15]. Moreover, plant can directly target endosymbionts to control behavior of its insect host. Recently, Chaudhary et al. demonstrated that during feeding, *B. aphidicola* in aphid saliva induced plant defense, which, in turn, reduced aphid fecundity [16]. Such interactions among aphid-endosymbiont-plant may indirectly influence virus transmission via modulation of plant networks [12]. However, limited information is currently available regarding whether aphid endosymbiont is involved in the non-persistent plant virus transmission. Our previous results demonstrated that CMV infection enhanced plant defense and reduced aphid fecundity [17]. Previously, we found that infection by CMV can shift feeding preference of its insect vector, *M. persicae*, from infected to healthy plants. Building on the preliminary research, we hypothesized that aphid endosymbiont *B. aphidicola* may affect herbivore behaviors through modulating plant volatile profiles.

To examine this hypothesis, we carried out the following objectives to investigate the 1) host preferences of viruliferous aphids between infected and healthy plants; 2) *B. aphidicola* abundance in CMV-infected and rifampicin-treated *M. persicae*, respectively; 3) volatile profiles of CMV-infected and non-infected host plants non-infested or infested by healthy, CMV-infected and rifampicin-treated *M. persicae*; and 4) functions of the resultant volatiles from Objective-3.

## Results

### Host preference of aphids between infected and healthy plants

**Fig. 1:**
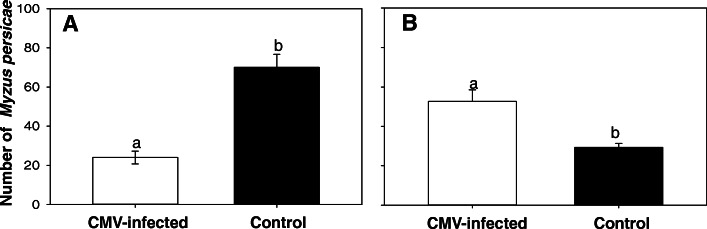
Preference of aphids between infected and healthy plants. **a**. CMV-infected aphids. **b**. non-infected aphids**.** Values are means ± SE. Different letters indicate significant differences (*p* < 0.05) by t-test

The number of *M. persicae* on CMV-infected plants was significantly lower than that on healthy plants (F_1, 16_ = 4.975, *P* < 0.001, Fig. 1a), while the number of non-viruliferous *M. persicae* on CMV-infected plants was significantly higher than that on healthy plants (F_1, 16_ = 2.602, *P* = 0.005, Fig. 1b), suggesting that CMV-infected aphids preferred healthy plants.

### *Buchnera aphidicola* abundance among CMV-infected and rifampicin-treated aphids

*Buchnera aphidicola* sequence, 1845-bp in length, shared 99.9% similarity to the *B. aphidicola* sequence from *M. persicae* genome (Accession number: CP002703, 275,749–276,779 site of *B. aphidicola* genome).

**Fig. 2:**
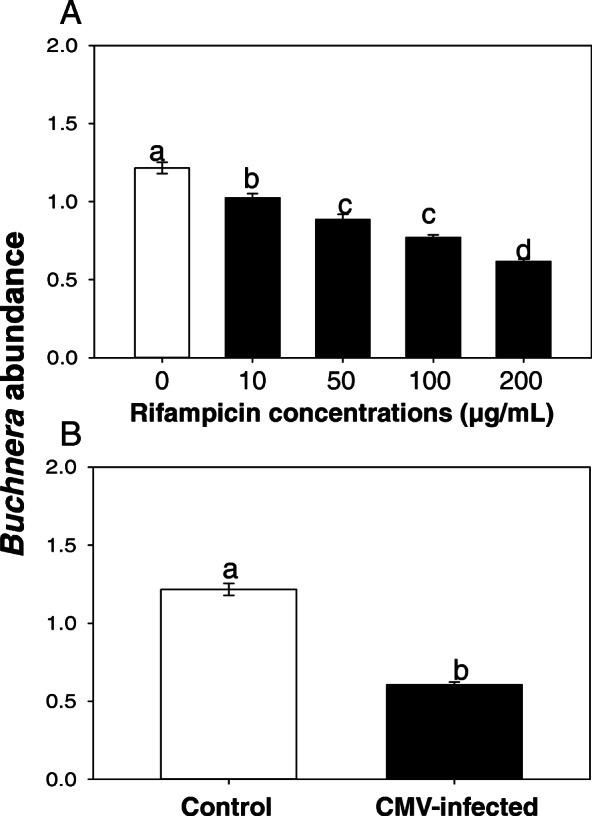
*Buchnera aphidicola* abundance of rifampicin-treated aphids. The index of *B. aphidicola* abundance, ratio of amplicons of *B. aphidicola Buch* gene/EF1α gene was determined. Values are means ± SE. Different letters indicate significant differences (*p* < 0.05) by ANOVA and Tukey’s HSD post hoc test

CMV infection significantly reduced the abundance of *B. aphidicola*, and the *Buchnera* abundance was significantly lower in CMV-infected aphids than in the control aphids (F_1, 4_ = 2.474, *P* < 0.001). Similarly, *B. aphidicola* was also significantly reduced after rifampicin treatment (F_4, 14_ = 71.708, *P* < 0.001), in a dose-dependent manner. In comparison to the controls, *B. aphidicola* abundance in *M. persicae* treated with 200 μg/mL rifampicin was reduced by > 2-fold (Fig. 2).

### Plant volatile profiles on plants infested by *M. persicae* with reduced *B. aphidicola* abundance

**Fig. 3:**
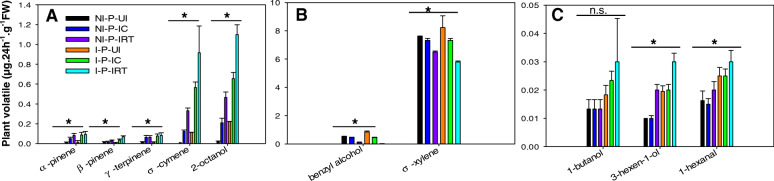
Content of plant volatiles in different treatment. NI-P-UI: non-infected control plants; NI-P-IC: non-infected plants infested by healthy aphids; NI-P-IRT: non-infected plants infested by rifampicin-treated aphids; I-P-UI: CMV-infected plants; I-P-IC: CMV-infected plants infested by healthy aphids; I-P-IRT: CMV-infected plants infested by rifampicin-treated aphids. **a**. Plant volatiles of α-pinene, β-pinene, γ-terpinene, σ-cymene, and 2-octanol, on plants in different treatments. **b**. Plant volatiles of benzyl alcohol and σ-xylene, on plants in different treatments. **c**. Plant volatiles of 1-butanol, 3-hexen-1-ol, and 1-hexanal, on plants in different treatments. Values are means ± SE. Asterisks indicate significant differences (*p* < 0.05) by ANOVA and Tukey’s HSD post hoc test

The volatile profiles were similar qualitatively, however, different quantitatively. Compared with healthy plants, healthy plants infested with healthy aphids released the significant higher titers of α-pinene, γ- terpinene; healthy plants infested with rifampicin-treated aphids released the significant higher titers of α-pinene, γ- terpinene, σ-cymene, and 2-octanol, while significant lower titers of benzyl alcohol and σ-xylene; CMV-infected plants significantly increased the titers of benzyl alcohol and σ-xylene, while significantly reduced the titers of σ-cymene and 2-octanol; CMV-infected plants infested with healthy aphids and CMV-infected plants infested with rifampicin-treated aphids both significantly increased the titers of α-pinene, β-pinene, γ- terpinene, σ-cymene and 2-octanol, while reduced the titers of benzyl alcohol and σ-xylene, significantly (Fig. 3).

### Function analysis of the resultant volatiles

**Fig. 4:**
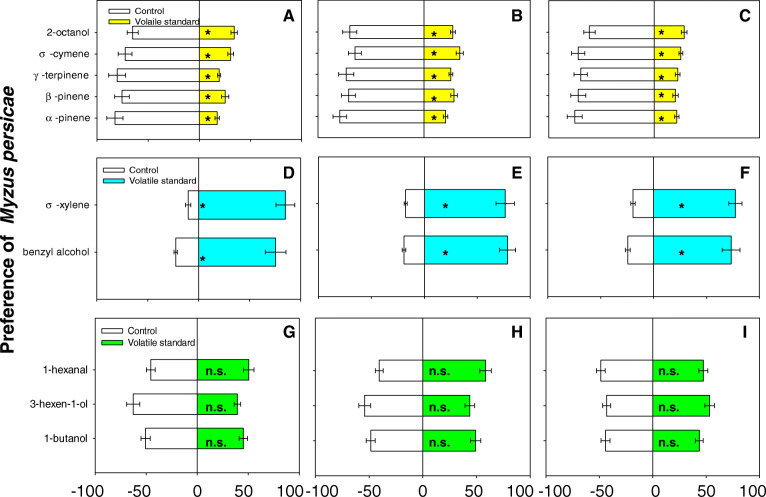
Aphid preference affected by volatiles. **a**. Healthy aphids’ preference. **b**. CMV-infected aphids’ preference. **c**. Rifampicin-treated aphids’ preference. **d**. Healthy aphids’ preference. **e**. CMV-infected aphids’ preference. **f**. Rifampicin-treated aphids’ preference. **g**. Healthy aphids’ preference. **h**. CMV-infected aphids’ preference. **i**. Rifampicin-treated aphids’ preference. Values are means ± SE. Asterisks indicate significant differences (*P* < 0.05) between volatile standard and control by a general linear model. n.s. indicates not significant between volatile standard and control

In Y-tube analysis, healthy, CMV-infected, and rifampicin-treated *M. persicae* were significantly repelled by 2-octanol, o-cymene, α-pinene, β-pinene, and γ-terpinene, while significantly attracted by benzyl alcohol and σ-xylene. As for 3-hexen-1-ol, 1-butanol, and n-hexanal, the healthy, CMV-infected, and rifampicin-treated *M. persicae* did not show any preference (Fig. 4).

## Discussion

Plant virus can influence the host selection behavior of its insect vector to facilitate the virus transmission [18, 19], such as the increased alate production in infected aphid vectors [17, 20]. Recently, the multi-trophic interactions among plant pathogens, insect vectors and plant hosts have expanded to involve a fourth party: bacterial symbionts harbored within the insect vector [12].

### Shift in feeding preference of CMV-infected aphids facilitates the virus transmission

It is expected that most plant viruses can induce changes in plants that have positive effects for transmission by insect vectors [21]. Here, CMV-infected aphids were attracted to the healthy plants rather than CMV-infected plants, and previous research on *Aphis gossypii* alates also showed the shift preference from CMV-infected to mock-inoculated plants [22]. Besides, when aphids were exposed to CMV-infected plants, the probing behavior showed a sharp change over time [22]. The increased number of CMV-infected aphids on healthy plants, in theory, will facilitate virus transmission in the field. However, the mechanism of aphid shifted preference mediated by non-persistent plant virus is largely unknown.

### CMV infection reduced the relative abundance of *B. aphidicola* in *M. persicae*

Our RT-qPCR analysis showed that CMV infection reduced the relative abundance of *B. aphidicola* in *M. persicae*. In a parallel experiment, rifampicin treatment, not surprisingly, significantly reduced the *B. aphidicola* titer in *M. persicae*, which is consistent with previous findings [23]. Although bacterial endosymbionts in *M. persicae* could be abundant and diverse, we only detected *B. aphidicola* following [24]. None of the other symbionts, such as *Rickettsia*, *Hamiltonella, Wolbachia,* and *Spiroplasma,* were detected in this study. Interestingly, however, the level of reduction in *B. aphidicola* abundance in *M. persicae* infected with CMV was comparable to the aphids treated with the highest concentration of antibiotics (200 μg/mL rifampicin; Fig. 2). Nevertheless, the eventual choice by aphids ultimately favor the transmission and spread of virus, an apparent incentive for virus during the co-evolution with its insect vector.

### Reduced *B. aphidicola* titer in aphids leads to the quantitative changes in plant volatiles

Plants often respond to herbivore attack by releasing a specific blend of volatiles [25]. Previous research showed that the volatiles emitted by CMV-infected plants appeared to be qualitatively similar to the blend emitted by healthy plants [8], which is in consistent with our results. Some terpenes typically repel arthropod herbivores, such as σ-cymene, α-pinene, β-pinene, and γ-terpinene [26–29], which is consistent with our results. σ-xylene, however, attracts aphids in our study, but deters whiteflies, *Bemisia tabaci* (Gennadius) [30]. The discrepancy might depend on insect species. Mauck et al. (2010) showed that aphids were attracted to CMV-infected plants at first, and the viruliferous aphids emigrated from infected plants at a higher rate and exhibited reduced population growth when forced to feed on infected plants, suggesting that reductions in host palatability lead to the rapid dispersal of CMV-infected aphids [31]. Here we found that CMV-infected plants released higher titer of attractive plant volatile, while after CMV-infected plants were infested by healthy aphids and *B. aphidicola*-decreased aphids, the repellent volatile was induced and the attractive volatiles were reduced, and the phenomena may also explain the rapid dispersal of CMV-infected aphids. The result might vary in field conditions. This effect may be indirect, because the higher attractivity of healthy plants could be likely induced just to limit the insect infection by viruses, since the viruliferous status hampers the aphid life cycle. For this reason, these aphids could be attracted by healthy plants just to escape further virus infection, and this may indirectly induce actual transmission.

**Fig. 5:**
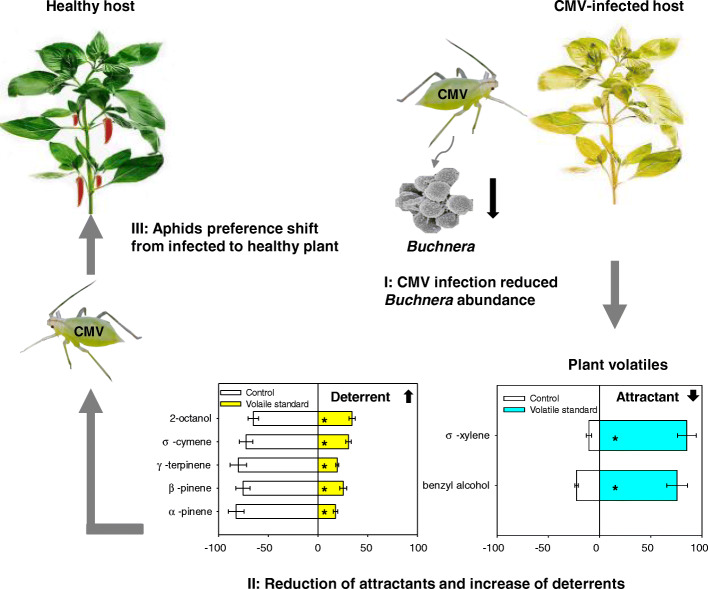
Proposed model for CMV outbreak facilitated by *B. aphidicola-*mediated shift in CMV-infected aphid’s feeding preference. Stage I: CMV-infected aphids prefer to feed on the healthy plants. Stage II: After feeding, the host plants are infected, and *B. aphidicola* abundance in CMV-infected aphids decreases. Stage III: After infection, the dynamics of plant volatile profiles is changed, with less attractants and more repellents. As a result, the feeding preference of aphid shifts from infected to healthy plants. Stage IV: Dispersal of CMV-infected aphids leads to CMV outbreak

The mutualism between aphid, *M. persicae,* and its primary symbiont, *B. aphidicola,* is obligate, in which the partners cannot survive without the other. By reducing the abundance of *B. aphidicola*, the overall fitness of *M. persicae* is compromised. CMV is exploiting the situation by presenting a choice to its insect vector between the immunocompromised (infected) and nutritionally intact (healthy) host plants. In the meantime, the reduced *B. aphidicola* abundance quantitatively changes the plant volatile profiles to orient a choice by the insect vector favoring the transmission and spread of virus (Fig. 5).

During the transmission process of plant virus by insect vectors in a non-persistent manner, plant viruses are believed to be retained in the insect stylet. The debate in this field is that whether insect endosymbionts are involved in the process of non-persistent virus transmission. Based on this study, we proposed a simple model for *B. aphidicola* in the role of CMV transmission. This model exemplified the dynamics of aphids’ preference shift from CMV-infected plants to non-infected plants. The *B. aphidicola* abundance decrease in viruliferous aphids affected the plant volatile profiles, which result in the aphid preference shift from infected to healthy plants, and finally leads to the CMV outbreak (Fig. 5).

## Conclusion and perspectives

Up to date, most attentions have been given to how insect endosymbionts regulate circulative virus transmission, and results show that *B. aphidicola* can protect luteovirids from degradation in the aphid hemolymph during virus transmission [32]. In this study, we found that *B. aphidicola* also contributes to the transmission of a non-persistent plant virus, in a different manner.

This is one of the first studies to empirically examine how endosymbionts of CMV-infected aphids impact dynamics of plant volatiles to manipulate aphid preference and CMV transmission. We testified that the symbiont is involved in non-persistent virus transmission. We conclude that the decrease of *B. aphidicola* abundance in CMV-infected aphids reduced the quantity of attractive volatile and increased the quantity of reject volatile to indirectly cause the dispersal of CMV-infected aphids. Endosymbiont-mediated volatile change will drive the viruliferous aphids to healthy plants, thus providing more opportunity for viruliferous aphids to transmit virus, which might be essential for CMV to evolve and adapt to a vector-borne lifestyle.

## Methods

### Host plant, aphid colonies and CMV culture

The green peach aphid, *Myzus persicae* (Sulzer), was a gift from Dr. Xiwu Gao of China Agricultural University and has been maintaining on the pepper plant, *Capsicum annuum* L., (Zhongjiao 5, Chinese Academy of Agricultural Sciences). The formal identification of the samples used in this study was performed by Xiao-Bin Shi. Voucher specimens were deposited in the herbarium of Hunan Plant Protection Institute. CMV-AN isolate (subgroup IB, stored at − 80 °C in *Nicotiana glutinosa*) was inoculated into pepper plants at the 4–5 true leaf stage [17]. Healthy pepper with the same developmental stage was used as the controls. After 9 days, virus infection was confirmed by double-sandwich enzyme-linked immunosorbent assay (DAS-ELISA) [17]. CMV-infected aphids were generated by allowing *M. persicae* to feed on CMV-infected plants for 5 min [33].

### Host preference of aphids between infected and healthy plants

The CMV-infected aphids and non-infected aphids were used in the preference test. The CMV-infected and healthy pepper plants were placed in two separate collection pots. The two arms of Y-tube olfactometer were connected to the two corresponding pots via vacuum lines. Individual aphids were released at the base of the Y-tube. Each aphid was observed no more than 20 min. A choice was recorded when the aphid moved > 10 cm onto either arm and stayed in that arm for at least 5 min, and a ‘no choice’ was recorded when the aphids were remained inactive during the testing period. After five aphids were tested, odor sources were switched to avoid any unpredictable asymmetry of the setup. A total number of 90 CMV-infected and non-infected aphids were tested for 3 times per day and for 3 days. The inner and outer arms of the Y-tube were carefully wiped with 95% ethanol and dried each day.

### The establishment of rifampicin-treated *M. persicae*, and quantification of *Buchnera aphidicola* in aphids

The rifampicin-treated *M. persicae* was established by maintaining aphids on artificial diets supplemented with rifampicin at concentrations of 10, 50, 100, and 200 μg/mL for 48 h [23, 34]. The control aphids were maintained on regular artificial diets for 48 h. After 48 h, *B. aphidicola* abundance was determined.

The genomic DNA of *Myzus persicae* was extracted with Chelex (Sigma-Aldrich, St Louis, MO, USA) following a protocol described previously [35]. Here, polymerase chain reaction (PCR)-based diagnostic tools were used to identify the endosymbionts in *M. persicae*, including *B. aphidicola*, *Rickettsia*, *Hamiltonella defensa*, *Wolbachia*, and *Spiroplasma* (Table 1). The resultant PCR products were cloned, sequenced, and confirmed using BLAST. Only the 1845 bp region of *B. aphidicola* was amplified and confirmed. The abundance of *B. aphidicola* in viruliferous and control aphids was estimated by the ratio of *B. aphidicola* amplicons of *Buch* (forward primer: AGCGGCCTCCTAAACGAAAA; reverse primer: AGTCGACATCGTTTACGGCA)/*EF1α* (forward primer: AGAATGGACAAACCCGTGAA; reverse primer: CACTGTATGGTGGTTCAGTAGAG) using RT-qPCR [36, 37]. For RT-qPCR analysis, three biological replicates were carried out for each experiment. For each biological replicate, four technical replicates were included. The relative expression of *Buch,* the target gene, was normalized to *EF1α,* the internal reference, using 2 − ΔΔCT method [38].

**Table 1:** Diagnostic primers used for the detection of endosymbionts in *M. persicae*

Endosymbiont	Primer sequence (5′-3′)^a^
*Buchnera aphidicola*	F: CATGGCTCAGATTCAACGCTGGCG R: CCCCTCGGTTACCTTCTTACGAC
*Wolbachia*	F: AGAGTTTGATCATGGCTCAGATTG R: TACCTTGTTACGACTTCACCCCAG
*Rickettsia*	F: AGAGTTTGATCMTGGCTCAG R: CATCCATCAGCGATAAATCTTTC
*Hamiltonella defensa*	F: AGCACAGTTTACTGAGTTCA R: TACGGYTACCTTGTTACGACTT
*Spiroplasma*	F: AGAGTTTGATCMTGGCTCAG R: TAGCCGTGGCTTTCTGGTAA

“^a^”: Forward and Reverse primers

### Plant volatile analysis

A headspace system (Shi et al., 2018) was used to collect volatiles, including healthy plants, healthy plants infested with control aphids, healthy plants infested with rifampicin-treated (200 μg/mL) aphids, CMV-infected plants, CMV-infected plants with control aphids, and CMV-infected plants infested with rifampicin-treated (200 μg/mL) aphids. Plant volatile of all the treatments were collected for 6 h, and aphid infestation was maintained in 6 h with clip-cages, and non-infestation plants were also treated with clip-cages. For each treatment, volatile collection was independently replicated three times, including three technical replicates for each biological replicate.

### Functional analysis of host plant volatiles

The response of healthy, CMV-infected, and rifampicin-treated *M. persicae* to the 10 detected volatiles was determined using a Y-tube olfactometer. The standards of 3-hexen-1-ol, 1-butanol, n-hexanal, benzyl alcohol, σ-xylene, 2-octanol, o-cymene, α-pinene, β-pinene and γ-terpinene were inlcuded in the Y-tube test. Two streams of purified air (filtered through activated charcoal) were led through two glass containers (a standard chemical and a purified air as the control) into the two arms of Y-tube olfactometer at 100 mL/min. The aphid preference was test according to the method described in the section of “Host preference of viruliferous aphids between infected and healthy plants”. For each treatment, a group of 90 healthy, CMV-infected, and rifampicin-treated aphids were tested for 3 times per day, for 3 days. A total of 2700 aphids were used to examine the function of the 10 resultant volatiles.

### Statistical analysis

SPSS version 20.0 (SPSS Inc., Chicago, IL, USA) was used for all statistical analyses. One sample t-test was used to compare the preference of CMV-infected aphids and to compare the *B. aphidicola* amplicons of *Buch* /EF1α in CMV-infected and control aphids. One-way ANOVA was used to compare the *B. aphidicola* amplicons of *Buch* /EF1α in rifampicin-treated aphids at concentrations of 0, 10, 50, 100, and 200 μg/mL. The volatiles released on plants in different treatments were also compared with one-way ANOVA. Effects of the 10 detected plant volatiles on preference of healthy, CMV-infected, and rifampicin-treated aphids in Y-tube olfactometer were compared with General linear model (GLM).

## Data Availability

The datasets used and/or analyzed during the current study are available from the corresponding author on reasonable request.

## Abbreviations

CMV: Cucumber mosaic virus
AGO1: ARGONAUTE1
DAS-ELISA: Double-sandwich enzyme-linked immunosorbent assay
